# The Barber Shop as a Menace to the Public Health

**Published:** 1898-06

**Authors:** A. Walter Suiter

**Affiliations:** Herkimer, N. Y., Examiner in physiology and hygiene, New York State Board of Medical Examiners.


### BUFFALO MEDICAL JOURNAL.

VOL. XXXVII. JUNE, 1898. NO. 11.

### Original Communications.

### THE BARBER SHOP AS A MENACE TO THE PUBLIC HEALTH.*

BY A. WALTER SUITER, A. M., M. D., Herkimer, N. Y.,
Examiner in physiology and hygiene, New York State Board of Medical Examiners.

IT SEEMS that the time has arrived when the attention of this association, and through it that of public health authorities in general, should be called to that part of personal hygiene which applies to the barber shop.

It is astonishing that while the sanitary relations of most, if not all, other public occupations have been investigated to the minutest detail, and salutary restrictions, in many instances of the most rigorous character, have been imposed to protect the public interest, the first attempt to regulate the operation of the barber shop and to apply to them the rules of sanitary administration has been made within the month just past.

That this common mode of infection should have for so long escaped authoritative attention in these days of asepsis and antisepsis may be noted as one of the most contradictory circumstances of our times.

To point out the necessities for reform in this respect will be the purpose of this brief paper, and the writer does not expect to do more than furnish a text for such remarks or such action on the part of the association as will tend to bring the subject properly to the notice of local health authorities, who alone have the power to act in such premises.

Historically, the barber has, from a very remote period of antiquity, held an important station among the occupations of the world.

---

* Read at the annual meeting of the American Public Health Association, Philadelphia, October 27, 1897.

He was, in a certain sense, the progenitor of the surgeon and the dentist of the present day and in ancient times was invested with professional rights and privileges by government sanction which might well excite the envy of his offsprings. To him was given the exclusive right to practise phlebotomy, which was until a somewhat recent date regarded as the most effective therapeutic agent known for the alleviation and cure of most of the diseases to which the human race is heir. Then, as now, the barber shop was a "focus of news" and "the favorite resort of idle persons," and the professional barber and hair dresser enjoyed the worldwide reputation for gossip and garrulity which is so familiar to us all.

By act of incorporation they were made "professors of the healing art" in England in 1461 during the reign of Edward IV., and so continued during the reign of George II. (A. D. 1726-1760), when the barber and surgeon were formally divorced.

We have some reason to believe that even in the remote periods mentioned, attention was given to the subject of sanitation in the selection and arrangement of the utensils made use of in their professional employment. Cleanliness was invariably provided for, a fact which is instanced by the symbol of the brass basin which has, for all time, been suspended from the striped pole which still constitutes the sign of the barber in many countries. This brass basin was always made with a crescent-shaped space cut into the side to receive a man's throat and thus prevent the soiling of clothes with the lather and other substances which were applied in the operation of shaving the beard. Again, it is worth noting that even in ancient Greece and Rome, barbers and *bathers* were associated in their labors and rivaled the physicians of that period in the practice of their art. Later on, in France, the "Society of Barbers and Bathers" was accorded the privilege of practising all sorts of surgery in spite of the protests of the regular surgeons. Barbering and bathing are to some extent associated in our time, but not to the extent comprehended by the subject of these remarks, it is a matter of regret to state.

If, as we presume, the sanitation of cleanliness was observed by the barber surgeons of medieval France, the system must have degenerated to the present date, for, as was above mentioned, the first attempt of modern times to regulate sanitarily the operations of the barber shop was recently made in the city of Paris. As a police regulation and by direction of the sanitary authorities, an order has been promulgated requiring that specific rules of asepsis and disinfection shall be observed in all shops for the protection of the public. These directions include the use of only metal combs, in order that they can be easily sterilised, and that, as far as possible, all instruments shall be of metal; that the razors, clippers, scissors, combs, and the like, shall be subjected to a heat of 100° centigrade before and after use; that the shaving brushes must be washed before and after use in boiling water and that the hands of the barbers shall be thoroughly cleansed before passing from one customer to another. They are required to use sterilised towels and pulverisers in place of the common powder puffs, and the hair that is removed is to be disinfected and promptly taken from the room. Chemical solutions are also prescribed for obvious purposes.

The writer is not informed as to the method by which it will be made certain that these regulations will be faithfully observed, but doubtless a system of inspection will be inaugurated under police surveillance which will insure detection in case of nonobservance and the proper punishment of all offenders.

When advised of the promulgation of this order by the Paris health department, the thought at once occurred that the same might well be universally adopted as an important reform in the matter of local sanitary administration. As individuals we are constantly reminded of the grave personal dangers which threaten the patron of the average barber shop and many of us have long since adopted measures of prevention for self-protection. The writer's experience does not differ from that of many others who have professional knowledge of the communicability of pediculi and disease. The magnificent (?) beard which he now presents to your view is the result of an occasion when a characteristic danger was imminent. One evening several years ago a sheepish-looking individual shuffled his way into my office waiting-room and requested a prescription. He was observed to be suffering from syphilis and presented a most unattractive appearance; his face was literally covered with eruptions and his mouth and lips were ulcerous in a high degree with the form of manifestation known as "mucous patches." His hair was fast falling and in short he was a perfect focus of infection. His case was disposed of and he was gladly dismissed.

Having to take an early morning train I shortly afterwards proceeded to the barber shop to prepare for my toilet. My barber's chair was occupied and I sat down to await my turn. As the occupant was about to arise I was startled to observe the very patient for whom I had prescribed an hour previous!

It is needless to say that I took my departure, although I was obliged to exercise some *finesse* in order to avoid exposing the professional secret that had been confided to me. Neither did I dare for the same reason to offer any suggestion for public protection. Instances with this latter feature, in this and other relations, are constantly occurring to physicians as all who listen will appreciate.

Then and there I resolved that my face should never again be shaved by a barber and the result is apparent. I also adopted a plan for my occasional visits to the barber shop for the trimming of hair and beard. Carrying always a packet containing shears, comb, brushes and clippers, I insist that everything be removed from the chair, clean towels be substituted and that the barber thoroughly wash his hands before proceeding; and for all this I tender him (beforehand if a stranger) a fee so large that he cannot complain of what ordinarily seems to him a hardship. The only inconvenience I experience is, that among the tonsorial fraternity of my vicinity I am compelled to bear the somewhat unsavory name of the "champion crank of the town."

This item of personal observation is introduced to direct attention to only one phase of the many dangerous situations which will readily occur to the sanitary mind, and it seems entirely unnecessary in this presence to attempt to elaborate this simple note of warning and protest by offering illustrative examples.

Instances almost without number might be collected and presented to prove the transmission of various diseases by the methods commonly practised in tonsorial operation. Pages might be written from the scientific standpoint to show the prevalent violation of well-established hygienic principles and the possibilities of parasitic and bacteriologic communication. Such an effort would be a work of supererogation indeed. The assumption is quite warrantable that there is scarcely a step in the processes usually employed by the barber which does not carry an element of danger when the most careful precautions are not observed. There is probably not a physician of considerable experience who could not cite examples which have occurred in his observation to support the argument presented, but it may well be doubted whether even with such precautions as might, and sometimes are, made use of in the ordinary way, such articles as combs, brushes and sponges would still be unclean and capable of conveying vitalised elementary substances from one person to another. Take the matter of sponges, for example. No surgeon thinks of using one a second time, unless it is passed through a process of disinfection requiring an amount of time and labor which more than covers the cost of a new one, and yet in many, if not most, barber shops the same sponge is repeatedly used from face to face, with nothing more than a simple squeezing between each sitter, which cannot remove the infected blood or pus from the often broken or eruptive cuticle.

In the opinion of the writer it is absolutely impossible, no matter how good may be the intention, to properly cleanse and sterilise any of the instruments employed without consuming an amount of time and material which would render such an effort impracticable. Hence it appears that nothing short of such compulsory regulations as have been adopted by the Paris authorities would be effective. Local administration of this character should be made to apply to every barber shop, whether rural or urban, and a system of inspection established to enforce the rules. There will be found in many localities some items which the Paris regulations do not seem to cover, such as the particularly dangerous use, from patron to patron, of the alum stick to staunch the flow of blood from the cut cuticle, the common use of the block of magnesia to complete the drying of the newly shaven skin, both of which articles sometimes do continuous service for weeks for the occupants of the same chair. Of course, these remarks do not apply to all shops, as many of them are kept as cleanly as is possible without scientific disinfection, and most of the better class of barbers would gladly join in the movement of reform if a proper opportunity were offered.

The legislature of the state of New York has had, for a year or two past, a proposition pending for the enactment of a law which would, in a great degree, serve to correct many of the evils referred to here. This bill emanates from a representative organisation of the barbers themselves and has the uniform support of all the better classes among them. They desire the law for self-protection and to elevate the general character of their business. By the provisions of the proposed regulation a board of examiners will be appointed, which will proceed to qualify the members of the trade by systematic examinations of such a nature as to demonstrate the fitness of each individual applying for a license and no one can practise except he is so qualified. It is understood that the knowledge of the scientific rules for disinfection and general sanitation will be an integral part of the requirements. This measure will be pushed during the coming session and the barbers' association will receive the support and active aid of the medical societies. Should it be enacted and health boards in general promulgate the rules of asepsis and disinfection by which to enforce the provisions of the law, nothing more can be desired.

It is understood that in Germany the general regulations for the licensing by examinations of all occupations in great part solves the problem of sanitation in barber shops, as only those are licensed who are especially qualified from all points of view. Such a system should be made applicable in all states and countries. Horseshoers are thus qualified in the state of New York and why is it not just as important to regulate barbers in the same manner?

It is remarkable that since this information has come to us from abroad the medical journals and newspapers are teeming with commendatory comments and wonder is often expressed why this subject has been for so long officially unnoticed. Without doubt the people will be quickly educated to the necessity for action and the efforts of health boards will be supplemented and facilitated by favorable public sentiment.

Let us adopt for our motto in this respect the Spanish proverb, "*Dicho y hecho*" (no quicker said than done) and with the greatest promptitude practicable demand that an intelligent and unprejudiced inquiry be made into this fruitful source of disease transmission, to the end that the fundamental principles of sanitary science may be made to apply to it as well as it does to many less threatening situations.

If the surgeon and the dentist are morally and, in a negative sense, legally, under obligations to disinfect and sterilise in all cases their instruments before using them, why is it not just as reasonable to hold a barber equally responsible for neglect of such precautions? And why should it not be properly within the province of health boards to protect the public in this regard and quite within the jurisdiction of this association to add its powerful influence towards the promotion of the means for the accomplishment of this much-to-be-desired reform? We are constantly striving to regulate the disinfection of railroad sleeping cars, passing ordinances to subvert the dangers of the spitting nuisance, loudly demanding the substitution of the individual for the common communion cup and in divers other fields are endeavoring to compel the proverbially heedless public to have a care for their own welfare, but it may well be said that the hygienic regulations of the barber shop far exceeds in importance the most of these undertakings.

